# Associations of CALLY, CLR, and CHR with all-cause mortality in patients receiving maintenance hemodialysis: a two-center retrospective cohort study

**DOI:** 10.3389/fendo.2026.1849614

**Published:** 2026-07-06

**Authors:** Ying Xu, Yujian He, Yuting Liu, Yanting Liang, Guanghao Wei, Zhen Wang, Nan Hu, Lijun Luo, Hualin Ma, Xinzhou Zhang

**Affiliations:** 1 Department of Hematology, The Second Clinical Medical College of Jinan University, The First Affiliated Hospital of Southern University of Science and Technology, Shenzhen People's Hospital, Shenzhen, Guangdong, China; 2 Department of Nephrology, The Second Clinical Medical College of Jinan University, The First Affiliated Hospital of Southern University of Science and Technology, Shenzhen People's Hospital, Shenzhen, Guangdong, China; 3 Shenzhen Key Laboratory of Kidney Diseases, Shenzhen, Guangdong, China; 4 Shenzhen Clinical Research Center for Geriatrics, Shenzhen People's Hospital, Shenzhen, Guangdong, China; 5 Department of Nephrology, Huidong People's Hospital, Huizhou, Guangdong, China

**Keywords:** all-cause mortality, CALLY index, C-reactive protein-to–high-density lipoprotein cholesterol ratio (CHR), C-reactive protein-to-lymphocyte ratio (CLR), maintenance hemodialysis

## Abstract

**Background:**

Patients undergoing maintenance hemodialysis (MHD) remain at high risk of death. Composite inflammation-related indices may capture inflammatory burden, immune status, nutritional reserve, and lipid-related protective capacity. This study evaluated the associations of the C-reactive protein-albumin-lymphocyte (CALLY) index, C-reactive protein-to-lymphocyte ratio (CLR), and C-reactive protein-to-high-density lipoprotein cholesterol ratio (CHR) with all-cause mortality in patients receiving MHD.

**Methods:**

This retrospective two-center cohort study included adult patients undergoing maintenance hemodialysis at Shenzhen People’s Hospital and Huidong People’s Hospital, China. Baseline data were collected between January 2017 and October 2022, and all patients were followed until death or October 2023. CALLY, CLR, and CHR were natural log-transformed and analyzed using Cox proportional hazards models, Kaplan-Meier curves, restricted cubic splines, subgroup analyses, time-dependent receiver operating characteristic curves, and sensitivity analyses after multiple imputation.

**Results:**

During a median follow-up of 36.67 months (IQR, 20.01–52.80 months), 125 patients died. In the fully adjusted Cox models, each one-unit increase in ln CALLY was associated with lower all-cause mortality risk (HR, 0.85; 95% CI, 0.77-0.94; P = 0.002), whereas ln CLR (HR, 1.19; 95% CI, 1.06-1.33; P = 0.003) and ln CHR (HR, 1.18; 95% CI, 1.07-1.31; P = 0.002) were associated with higher mortality risk. Kaplan-Meier curves showed significant survival differences across tertiles (log-rank P = 0.016, 0.012, and 0.044). Restricted cubic spline analyses showed significant overall associations (P = 0.0158, 0.0138, and 0.0301) without significant nonlinearity. The 3-year AUCs were 0.603, 0.598, and 0.563 for ln CALLY, ln CLR, and ln CHR, respectively. Sensitivity analyses yielded similar Cox regression results.

**Conclusions:**

ln CALLY, ln CLR, and ln CHR were independently associated with all-cause mortality in MHD patients and may serve as accessible markers for initial prognostic risk stratification.

## 1 Introduction

Hemodialysis (HD) is one of the most widely used renal replacement therapies for patients with end-stage kidney disease (ESKD). By removing metabolic waste products and excess fluid through diffusion, convection, and ultrafiltration across a semipermeable membrane, HD helps maintain fluid, electrolyte, and acid-base homeostasis (1). With the increasing burden of chronic kidney disease and broader access to renal replacement therapy, the number of patients receiving maintenance hemodialysis (MHD) has continued to rise (1). Despite advances in dialysis technology and clinical management, long-term outcomes in this population remain unsatisfactory, and mortality remains a major clinical concern.

Poor prognosis in patients undergoing MHD is not driven by a single pathway. Rather, it reflects the cumulative effects of multiple interrelated biological disturbances (2–4). Chronic inflammation plays a central role and is closely associated with cardiovascular events, infection, protein-energy wasting, and death. Persistent low-grade inflammation in this setting may arise from the uremic milieu, oxidative stress, recurrent infection, vascular access-related factors, and repeated exposure to dialysis-related materials (2, 5). Immune dysfunction and metabolic abnormalities frequently coexist, further contributing to adverse outcomes (3, 4). Because these processes often overlap and amplify one another, conventional single biomarkers may be insufficient for comprehensive risk evaluation in patients undergoing MHD.

In recent years, increasing attention has been paid to inflammation-related indices derived from routine laboratory parameters (6–8). Compared with isolated biomarkers, these composite indices may better reflect the complex biological status of patients by integrating information from different but related domains. The C-reactive protein-to-lymphocyte ratio (CLR) combines systemic inflammation with immune status, whereas the C-reactive protein-to-high-density lipoprotein cholesterol ratio (CHR) reflects the interaction between inflammatory burden and lipid-related protective capacity (6, 7). The C-reactive protein-albumin-lymphocyte (CALLY) index further incorporates nutritional reserve into this framework (8). Because CRP, lymphocyte count, albumin, and HDL-C are readily available in routine practice, these indices may provide simple and clinically meaningful tools for prognostic stratification.

Although several inflammation-related indices have shown prognostic value in different disease settings, evidence in the hemodialysis population remains limited, particularly regarding mortality outcomes. More importantly, few studies have evaluated these indices in parallel within the same MHD cohort. Given the high prevalence of chronic inflammation, immune dysregulation, lipid metabolic disturbance, and nutritional impairment in patients receiving MHD, a comparative assessment of these indices may provide additional insight into risk stratification. Therefore, this study aimed to investigate the associations of log-transformed CALLY, CLR, and CHR with all-cause mortality in patients undergoing MHD and to evaluate these associations using conventional survival analyses, time-dependent ROC analyses, and sensitivity analyses.

## 2 Methods

### 2.1 Study design and participants

This retrospective cohort study included patients who underwent maintenance hemodialysis (MHD) at the blood purification centers of Shenzhen People’s Hospital and Huidong People’s Hospital, China. The baseline data collection period was from January 2017 to October 2022. All eligible patients were followed from cohort entry until death or the administrative censoring date in October 2023. ESKD was defined as irreversible kidney failure requiring long-term renal replacement therapy, and all included patients had received maintenance hemodialysis for at least 3 months. A total of 798 hemodialysis patients were initially screened, including 486 from Shenzhen People’s Hospital and 312 from Huidong People’s Hospital.

**Figure 1:**
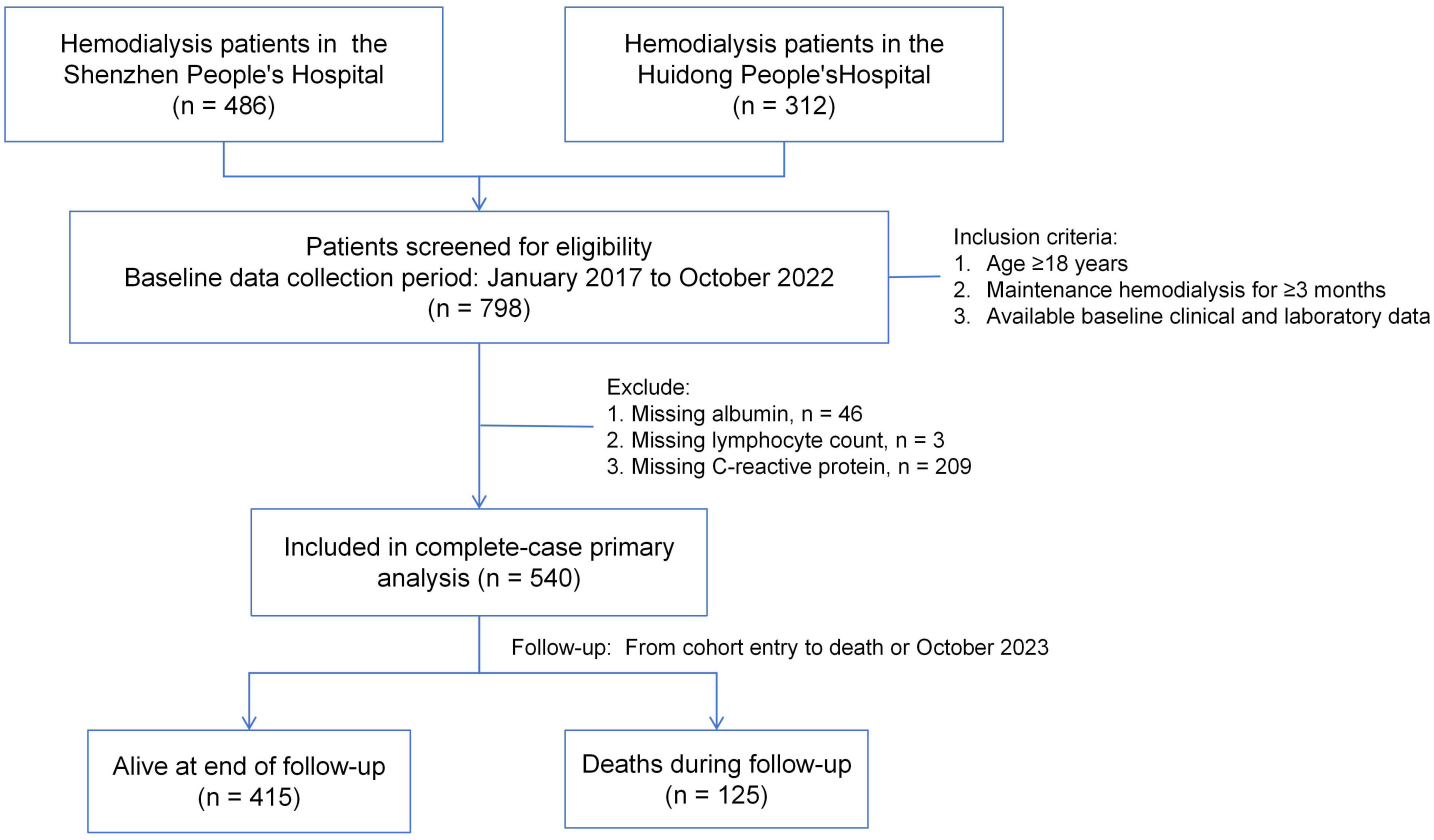
Flowchart of patient selection in the study cohort.

The inclusion criteria were as follows: (1) age ≥ 18 years; (2) receipt of MHD for at least 3 months; and (3) availability of baseline demographic information, clinical records, and relevant examination data. For the complete-case primary analysis, patients with missing variables required for calculating inflammation-related indices were excluded, including 46 patients with missing albumin data, 3 with missing lymphocyte count data, and 209 with missing C-reactive protein (CRP) data. Finally, 540 patients were included in the primary analysis. All patients were followed until death or October 2023, and all-cause mortality and follow-up time were recorded (**Figure 1**).

### 2.2 Data collection

Baseline data were obtained from the electronic medical record systems and information management systems of the two participating centers. Baseline clinical and laboratory data were defined as the first available records obtained at cohort entry before outcome follow-up began. The collected variables included demographic characteristics, cause of ESKD, comorbidities, and laboratory parameters.

Demographic and clinical variables included hospital source, age, sex, dialysis vintage, cause of ESKD, hypertension, diabetes, coronary heart disease, congestive heart failure, and history of stroke. Causes of ESKD were classified as diabetic nephropathy, hypertensive nephropathy, primary glomerulonephritis, and other causes.

Baseline laboratory measurements included white blood cell count (WBC), red blood cell count (RBC), hemoglobin (HB), platelet count (PLT), lymphocyte count (LYM), albumin (ALB), CRP, and high-density lipoprotein cholesterol (HDL-C). Laboratory measurements at both centers were performed according to standardized testing protocols and quality-control procedures required for tertiary hospital laboratories, ensuring consistency of laboratory data across centers.

### 2.3 Definition of inflammation-related indices

Three inflammation-related indices were evaluated: the C-reactive protein-albumin-lymphocyte (CALLY) index, the CRP-to-lymphocyte ratio (CLR), and the CRP-to-HDL-C ratio (CHR). The indices were calculated as follows: CALLY = ALB (g/L) × LYM (10^9/L)/[CRP (mg/L) × 10]; CLR = CRP (mg/L)/LYM (10^9/L); and CHR = CRP (mg/L)/HDL-C (mmol/L).

Because CALLY, CLR, and CHR were skewed, all three indices were natural log-transformed before subsequent analyses: ln CALLY = log(CALLY), ln CLR = log(CLR), and ln CHR = log(CHR). Each log-transformed index was analyzed as both a continuous variable and a categorical variable. For categorical analyses, patients were divided into tertiles according to the distribution of each log-transformed index, with the lowest tertile used as the reference group.

### 2.4 Statistical analysis

All statistical analyses were performed using R software (version 4.4.1). The main R packages used included survival, survminer, rms, mice, timeROC, pROC, readr, and patchwork. A two-sided P value <0.05 was considered statistically significant. Baseline characteristics were compared between patients who died during follow-up and those who remained alive. Continuous variables were expressed as mean ± standard deviation (SD) or median and interquartile range (IQR), as appropriate. Categorical variables were expressed as frequencies and percentages. Between-group comparisons were performed using the independent-samples t test, Mann-Whitney U test, chi-square test, or Fisher’s exact test, as appropriate.

The associations of ln CALLY, ln CLR, and ln CHR with all-cause mortality were evaluated using Cox proportional hazards regression models, and hazard ratios (HRs) with 95% confidence intervals (CIs) were calculated. The crude model was unadjusted. Model 1 adjusted for age, sex, dialysis vintage, and cause of ESKD. Model 2 further adjusted for hypertension, diabetes, coronary heart disease, congestive heart failure, and history of stroke. Model 3 additionally adjusted for WBC, RBC, HB, and PLT. Tertile categories of each log-transformed index were entered as ordinal variables to test for linear trends. To avoid overadjustment and collinearity, CRP, lymphocyte count, albumin, and HDL-C were not additionally included in the multivariable models when the corresponding composite index was entered as the exposure variable.

Kaplan-Meier survival curves were plotted according to tertiles of ln CALLY, ln CLR, and ln CHR, and differences among groups were compared using the log-rank test. Restricted cubic spline analyses were performed to assess dose-response relationships and potential nonlinearity. Subgroup analyses were conducted according to hospital source, sex, cause of ESKD, hypertension, diabetes, coronary heart disease, congestive heart failure, and history of stroke. Effect modification was assessed using interaction terms.

Time-dependent ROC analyses were used to evaluate the discriminatory performance of ln CALLY, ln CLR, and ln CHR for all-cause mortality at 1, 3, and 5 years. AUCs with 95% CIs, best thresholds, sensitivity, specificity, and Youden indexes were calculated. Pairwise DeLong-type tests based on the iid representation of the AUC estimator were used to compare the AUCs among the three indices. Crude Cox model-based time-dependent AUC curves were also generated across follow-up.

To assess the robustness of the primary findings, multiple imputation was performed for missing values, and the full screened cohort of 798 patients was used for sensitivity analyses. Cox regression analyses were repeated in the imputed dataset using the same model structure. Center-specific Cox regression analyses were also performed separately in Shenzhen People’s Hospital and Huidong People’s Hospital.

## 3 Results

### 3.1 Baseline characteristics

As shown in **Table 1**, a total of 540 patients undergoing MHD were included in the complete-case primary analysis. During a median follow-up of 36.67 months (IQR, 20.01–52.80 months), 125 patients died and 415 remained alive. The mean age of the overall population was 60.59 ± 14.32 years, and 62.59% of patients were male. Compared with survivors, patients who died were older (69.85 ± 12.19 vs. 57.80 ± 13.74 years, P < 0.0001). Dialysis vintage and sex distribution did not differ significantly between groups. Hospital source differed by survival status, with a higher proportion of patients from Huidong People’s Hospital among non-survivors (61.60% vs. 47.71%, P < 0.01).

**Table 1:** Baseline characteristics of maintenance hemodialysis patients stratified by survival status.

Variable	Total (n=540)	Survivor (n=415)	Non-survivor (n=125)	P
Hospital				<0.01
Huidong	275(50.93)	198(47.71)	77(61.60)	
Shenzhen	265(49.07)	217(52.29)	48(38.40)	
Sex				0.71
Female	202(37.41)	153(36.87)	49(39.20)	
Male	338(62.59)	262(63.13)	76(60.80)	
Age	60.59 ± 14.32	57.80 ± 13.74	69.85 ± 12.19	<0.0001
Dialysis age (months)	39.99 ± 54.04	40.25 ± 51.51	39.14 ± 61.93	0.86
During time (months)	37.90 ± 22.39	43.36 ± 19.96	19.77 ± 20.44	<0.0001
Cause of ESKD				<0.01
Diabetic Nephropathy	273(50.56)	203(48.92)	70(56.00)	
Primary glomerulonephritis	144(26.67)	123(29.64)	21(16.80)	
Hypertensive nephropathy	80(14.81)	52(12.53)	28(22.40)	
Other	43(7.96)	37(8.92)	6(4.80)	
Hypertension				0.01
No	121(22.41)	104(25.06)	17(13.60)	
Yes	419(77.59)	311(74.94)	108(86.40)	
Diabetes				0.05
No	353(65.37)	281(67.71)	72(57.60)	
Yes	187(34.63)	134(32.29)	53(42.40)	
Coronary heart disease				<0.0001
No	387(71.67)	316(76.14)	71(56.80)	
Yes	153(28.33)	99(23.86)	54(43.20)	
Congestive heart failure				<0.01
No	514(95.19)	402(96.87)	112(89.60)	
Yes	26(4.81)	13(3.13)	13(10.40)	
Cerebral apoplexy history				<0.01
No	464(85.93)	367(88.43)	97(77.60)	
Yes	76(14.07)	48(11.57)	28(22.40)	
WBC	6.99 ± 2.82	7.01 ± 2.82	6.92 ± 2.81	0.75
RBC	3.46 ± 0.82	3.47 ± 0.84	3.43 ± 0.76	0.68
HB	97.87 ± 21.50	98.06 ± 22.27	97.23 ± 18.80	0.68
PLT	203.25 ± 69.90	203.09 ± 69.41	203.76 ± 71.76	0.93
LYM	1.23 ± 0.54	1.27 ± 0.55	1.10 ± 0.50	<0.001
ALB	37.46 ± 5.18	37.76 ± 5.34	36.46 ± 4.51	<0.01
CRP	3.39 (0.94, 11.59)	3.01 (0.79, 9.84)	5.09 (1.75, 18.86)	0.001
HDL-C	1.02 ± 0.34	1.02 ± 0.33	0.99 ± 0.38	0.44
Composite inflammation-related indices				
CALLY	1.34 (0.35, 4.45)	0.69 (0.15, 2.76)	1.55 (0.45, 5.23)	<0.001
ln CALLY	0.29 (-1.06, 1.49)	0.44 (-0.80, 1.65)	-0.38 (-1.92, 1.02)	<0.001
ln CALLY Group				<0.01
Q1	180(33.33)	123(29.64)	57(45.60)	
Q2	180(33.33)	144(34.70)	36(28.80)	
Q3	180(33.33)	148(35.66)	32(25.60)	
CHR	3.67 (0.87, 12.24)	3.16 (0.76, 10.87)	6.14 (1.69, 23.16)	<0.001
ln CHR	1.30 (-0.14, 2.50)	1.15 (-0.28, 2.39)	1.81 (0.52, 3.14)	<0.001
ln CHR Group				<0.01
Q1	180(33.33)	149(35.90)	31(24.80)	
Q2	180(33.33)	141(33.98)	39(31.20)	
Q3	180(33.33)	125(30.12)	55(44.00)	
CLR	2.98 (0.83, 10.56)	2.42 (0.71, 8.34)	5.59 (1.31, 22.80)	<0.001
ln CLR	1.09 (-0.19, 2.36)	0.89 (-0.35, 2.12)	1.72 (0.27, 3.13)	<0.001
ln CLR Group				<0.01
Q1	180(33.33)	147(35.42)	33(26.40)	
Q2	180(33.33)	146(35.18)	34(27.20)	
Q3	180(33.33)	122(29.40)	58(46.40)	

ESKD, End-Stage Renal Disease; WBC, white blood cell; RBC, Red Blood Cell; HB, Hemoglobin; PLT, Platelets; LYM, Lymphocyte; ALB, Albumin; CRP, C-Reactive Protein; HDL-C, High-Density Lipoprotein Cholesterol; CALLY, C-reactive protein to Albumin Lymphocyte ratio; CLR, CRP to Lymphocyte ratio; CHR, CRP to HDL-C ratio.

Several baseline clinical characteristics differed by survival status. The distribution of causes of ESKD differed significantly between patients who died and those who survived (P < 0.01). Diabetic nephropathy and hypertensive nephropathy were more frequent among patients who died, whereas primary glomerulonephritis was more frequent among survivors. Hypertension was more common among patients who died than among survivors (86.40% vs. 74.94%, P = 0.01). Diabetes was also more common among patients who died (42.40% vs. 32.29%, P = 0.05). Coronary heart disease, congestive heart failure, and history of stroke were more prevalent among patients who died (all P < 0.01).

Regarding laboratory parameters, lymphocyte count and albumin were significantly lower in patients who died than in survivors (1.10 ± 0.50 vs. 1.27 ± 0.55, P < 0.001; 36.46 ± 4.51 vs. 37.76 ± 5.34, P < 0.01, respectively), whereas CRP was significantly higher (5.09 [1.75, 18.86] vs. 3.01 [0.79, 9.84], P = 0.001). No significant differences were observed in WBC, RBC, HB, PLT, or HDL-C. Among the composite inflammation-related indices, patients who died had lower ln CALLY values (-0.38 [-1.92, 1.02] vs. 0.44 [-0.80, 1.65], P < 0.001) and higher ln CLR (1.72 [0.27, 3.13] vs. 0.89 [-0.35, 2.12], P < 0.001) and ln CHR values (1.81 [0.52, 3.14] vs. 1.15 [-0.28, 2.39], P < 0.001). The distributions of ln CALLY, ln CLR, and ln CHR tertiles also differed significantly by survival status. Deaths were more frequent in the lowest tertile of ln CALLY and in the highest tertiles of ln CLR and ln CHR.

### 3.2 Associations of log-transformed inflammation-related indices with all-cause mortality

Cox proportional hazards regression models were used to evaluate the associations of ln CALLY, ln CLR, and ln CHR with all-cause mortality (**Table 2**). For ln CALLY, higher continuous values were associated with lower mortality risk in all models. In the fully adjusted Model 3, each one-unit increase in ln CALLY was associated with lower all-cause mortality risk (HR, 0.85; 95% CI, 0.77-0.94; P = 0.002). Compared with the lowest tertile, the middle and highest tertiles were associated with lower mortality risk, with HRs of 0.59 (95% CI, 0.38-0.91; P = 0.02) and 0.56 (95% CI, 0.35-0.89; P = 0.01), respectively. The trend across tertiles was significant (P for trend = 0.01).

**Table 2:** Cox regression analyses of associations between log-transformed inflammation-related indices and all-cause mortality in maintenance hemodialysis patients.

Character	Crude model		Model 1		Model 2		Model 3	
	95%CI	P	95%CI	P	95%CI	P	95%CI	P
ln CALLY	0.85(0.78, 0.93)	<0.001	0.89(0.81, 0.97)	0.01	0.89(0.81, 0.97)	0.01	0.85(0.77, 0.94)	0.002
ln CALLY group								
Q1	ref		ref		ref		ref	
Q2	0.64(0.42, 0.97)	0.04	0.69(0.45, 1.05)	0.08	0.66(0.43, 1.02)	0.06	0.59(0.38, 0.91)	0.02
Q3	0.57(0.37, 0.88)	0.01	0.64(0.41, 0.99)	0.05	0.64(0.41, 1.00)	0.05	0.56(0.35, 0.89)	0.01
p for trend		0.01		0.04		0.04		0.01
ln CLR	1.15(1.04, 1.27)	0.005	1.12(1.01, 1.24)	0.03	1.13(1.02, 1.25)	0.02	1.19(1.06, 1.33)	0.003
ln CLR group								
Q1	ref		ref		ref		ref	
Q2	1.29(0.80, 2.07)	0.29	1.35(0.84, 2.18)	0.21	1.31(0.81, 2.11)	0.27	1.37(0.84, 2.22)	0.21
Q3	1.73(1.11, 2.68)	0.02	1.6(1.02, 2.52)	0.04	1.66(1.05, 2.62)	0.03	1.97(1.22, 3.20)	0.01
p for trend		0.01		0.04		0.03		0.01
ln CHR	1.17(1.07, 1.29)	<0.001	1.13(1.03, 1.25)	0.01	1.14(1.03, 1.25)	0.01	1.18(1.07, 1.31)	0.002
ln CHR group								
Q1	ref		ref		ref		ref	
Q2	1.01(0.63, 1.63)	0.96	1(0.62, 1.62)	1.00	0.95(0.59, 1.55)	0.85	0.96(0.59, 1.56)	0.86
Q3	1.7(1.11, 2.62)	0.01	1.59(1.02, 2.47)	0.04	1.58(1.02, 2.45)	0.04	1.8(1.13, 2.84)	0.01
p for trend		0.01		0.03		0.03		0.01

Crude model: Composite Inflammation-Related Indices.

Model 1: Crude model further adjusted age, Sex, Dialysis age, Cause of ESKD.

Model 2: Crude model further adjusted age, Sex, Dialysis age, Cause of ESKD, Hypertension, Diabetes, Coronary heart disease, Congestive heart failure, Cerebral apoplexy history.

Model 3: Crude model further adjusted age, Sex, Dialysis age, Cause of ESKD, Hypertension, Diabetes, Coronary heart disease, Congestive heart failure, Cerebral apoplexy history, WBC, RBC, HB, PLT.

For ln CLR, higher continuous values were associated with increased all-cause mortality risk in all models. In Model 3, the HR per one-unit increase in ln CLR was 1.19 (95% CI, 1.06-1.33; P = 0.003). Compared with the lowest tertile, the middle tertile was not significantly associated with mortality (HR, 1.37; 95% CI, 0.84-2.22; P = 0.21), whereas the highest tertile was associated with higher mortality risk (HR, 1.97; 95% CI, 1.22-3.20; P = 0.01). The trend across tertiles was significant (P for trend = 0.01).

For ln CHR, higher continuous values were also associated with increased all-cause mortality risk. In Model 3, the HR per one-unit increase in ln CHR was 1.18 (95% CI, 1.07-1.31; P = 0.002). Compared with the lowest tertile, the middle tertile was not significantly associated with mortality (HR, 0.96; 95% CI, 0.59-1.56; P = 0.86), whereas the highest tertile was associated with higher mortality risk (HR, 1.80; 95% CI, 1.13-2.84; P = 0.01). The trend across tertiles was significant (P for trend = 0.01).

### 3.3 Kaplan-Meier survival analysis

**Figure 2:**
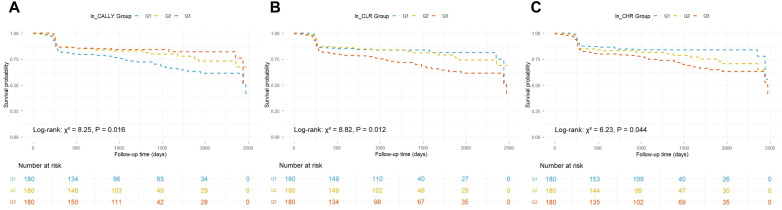
Kaplan–Meier survival curves for all-cause mortality according to tertiles of log-transformed inflammation-related indices. **(A)** ln CALLY, **(B)** ln CLR, and **(C)** ln CHR.

Kaplan-Meier survival curves showed significant differences in cumulative survival across tertiles of all three log-transformed indices. For ln CALLY, patients in Q1 had the lowest survival probability, whereas those in Q3 had the highest survival probability (log-rank χ² = 8.25, P = 0.016; **Figure 2A**). For ln CLR, patients in Q3 had the poorest survival, whereas those in Q1 had the most favorable survival (log-rank χ² = 8.82, P = 0.012; **Figure 2B**). For ln CHR, survival probability also decreased across increasing tertiles, with the poorest cumulative survival observed in Q3 (log-rank χ² = 6.23, P = 0.044; **Figure 2C**).

### 3.4 Restricted cubic spline analyses

**Figure 3:**
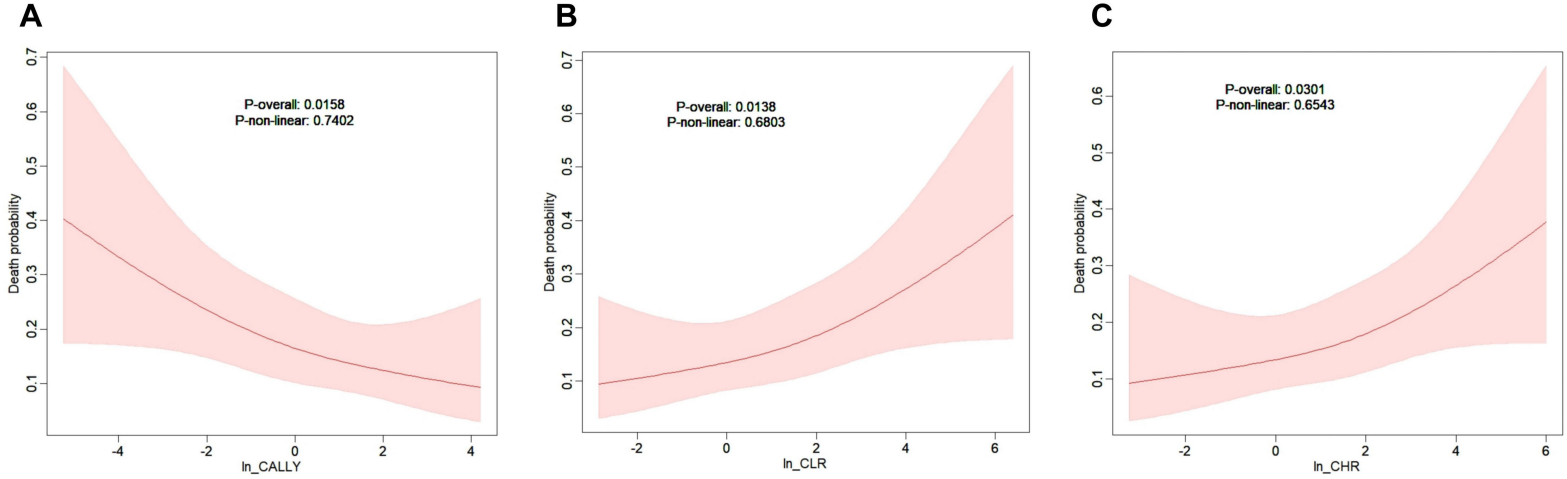
Restricted cubic spline analyses of the associations between log-transformed inflammation-related indices and death probability. **(A)** ln CALLY, **(B)** ln CLR, and **(C)** ln CHR. Solid lines represent the fitted associations, and shaded areas represent 95% confidence intervals. P values for overall association and nonlinearity are shown in each panel.

Restricted cubic spline analyses were performed to assess dose-response relationships between the log-transformed inflammation-related indices and all-cause mortality. ln CALLY showed a significant overall association with mortality risk (P for overall = 0.0158), with no evidence of nonlinearity (P for nonlinearity = 0.7402; **Figure 3A**).

ln CLR was also significantly associated with mortality risk (P for overall = 0.0138), with no significant nonlinear component (P for nonlinearity = 0.6803; **Figure 3B**). Similarly, ln CHR showed a significant overall association with mortality risk (P for overall = 0.0301), without evidence of nonlinearity (P for nonlinearity = 0.6543; **Figure 3C**).

### 3.5 Subgroup analyses

**Figure 4:**
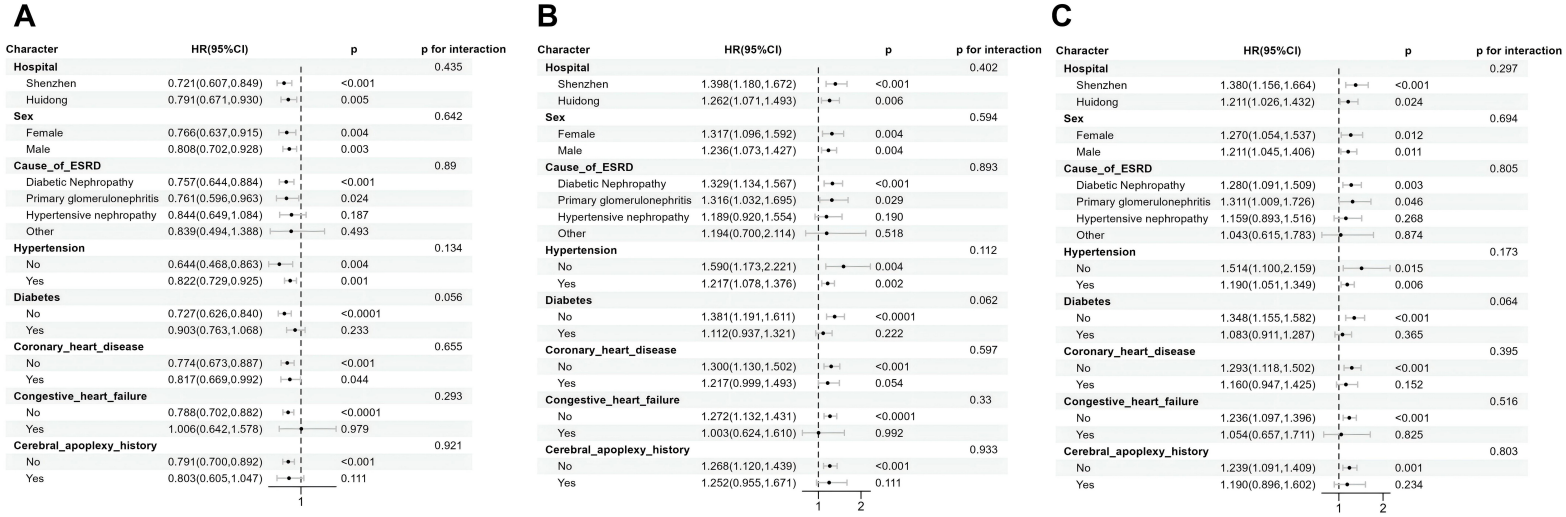
Subgroup analyses of the associations between log-transformed inflammation-related indices and all-cause mortality. **(A)** ln CALLY, **(B)** ln CLR, and **(C)** ln CHR. Hazard ratios and 95% confidence intervals are shown across prespecified subgroups. The dashed vertical line indicates a hazard ratio of 1. P values for interaction were calculated to assess effect modification across subgroups.

Subgroup analyses were performed across hospital source, sex, cause of ESKD, hypertension, diabetes, coronary heart disease, congestive heart failure, and history of stroke (**Figure 4**). The association between ln CALLY and all-cause mortality was assessed in each subgroup. No significant interaction was observed for hospital source, sex, cause of ESKD, hypertension, diabetes, coronary heart disease, congestive heart failure, or history of stroke (all P for interaction >0.05; **Figure 4A**).

The associations of ln CLR and ln CHR with all-cause mortality were also evaluated across the same prespecified subgroups. No significant interaction was detected for either index across the subgroup variables (all P for interaction >0.05; **Figures 4B, C**).

### 3.6 Time-dependent ROC analyses

**Figure 5:**
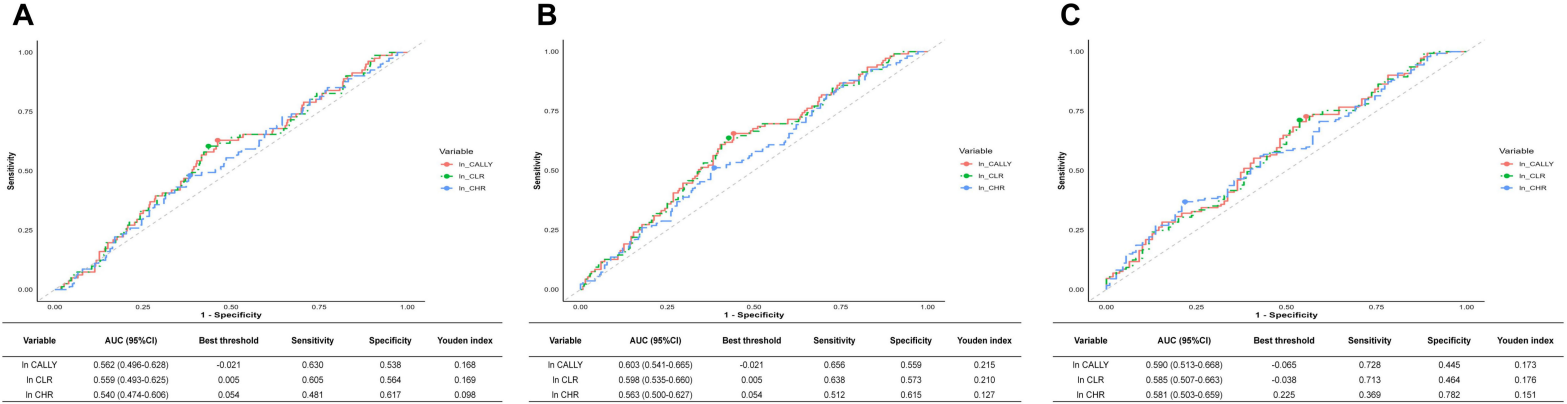
Time-dependent receiver operating characteristic curves for ln CALLY, ln CLR, and ln CHR in predicting all-cause mortality. **(A)** 1-year, **(B)** 3-year, and **(C)** 5-year prediction. The tables below each panel show the AUC with 95% confidence interval, optimal cutoff value, sensitivity, specificity, and Youden index for each index.

Time-dependent ROC analyses were performed for ln CALLY, ln CLR, and ln CHR at 1, 3, and 5 years (**Figure 5**). At 1 year, the AUCs were 0.562 (95% CI, 0.496-0.628) for ln CALLY, 0.559 (95% CI, 0.493-0.625) for ln CLR, and 0.540 (95% CI, 0.474-0.606) for ln CHR. At 3 years, the AUCs were 0.603 (95% CI, 0.541-0.665), 0.598 (95% CI, 0.535-0.660), and 0.563 (95% CI, 0.500-0.627), respectively. At 5 years, the AUCs were 0.590 (95% CI, 0.513-0.668), 0.585 (95% CI, 0.507-0.663), and 0.581 (95% CI, 0.503-0.659), respectively.

The best thresholds for ln CALLY, ln CLR, and ln CHR were -0.021, 0.005, and 0.054 at 1 year; -0.021, 0.005, and 0.054 at 3 years; and -0.065, -0.038, and 0.225 at 5 years, respectively. The corresponding sensitivity and specificity values were 0.630 and 0.538 for ln CALLY, 0.605 and 0.564 for ln CLR, and 0.481 and 0.617 for ln CHR at 1 year; 0.656 and 0.559, 0.638 and 0.573, and 0.512 and 0.615 at 3 years; and 0.728 and 0.445, 0.713 and 0.464, and 0.369 and 0.782 at 5 years, respectively.

**Figure 6:**
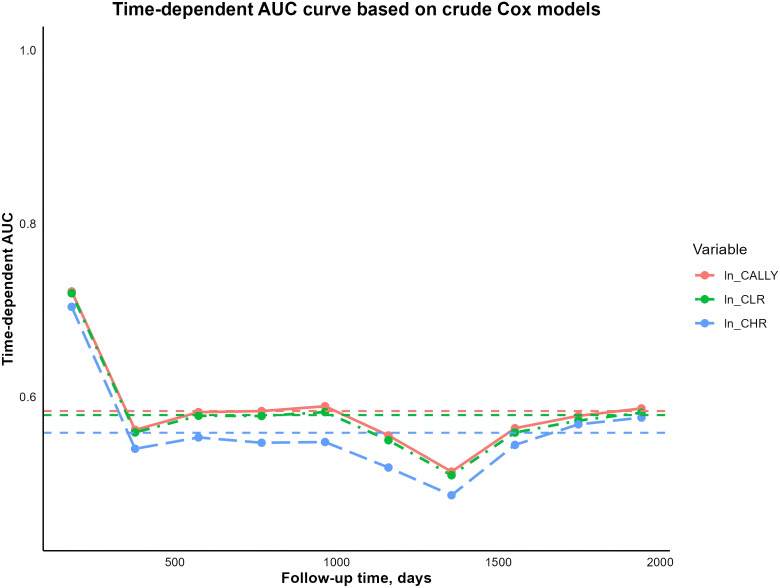
Time-dependent AUC curves based on crude Cox models for ln CALLY, ln CLR, and ln CHR. Curves show changes in time-dependent AUC values across follow-up time. Dashed horizontal lines indicate the corresponding reference AUC levels for each index.

Pairwise DeLong-type tests showed no significant differences among the three indices at 1 year (P = 0.226 for ln CALLY vs. ln CLR, P = 0.110 for ln CALLY vs. ln CHR, and P = 0.163 for ln CLR vs. ln CHR) or 5 years (P = 0.096, P = 0.495, and P = 0.761, respectively). At 3 years, significant pairwise differences were observed for ln CALLY vs. ln CLR (P = 0.018), ln CALLY vs. ln CHR (P = 0.002), and ln CLR vs. ln CHR (P = 0.006; **Table 3**). Crude Cox model-based time-dependent AUC curves showed time-varying and modest discrimination across follow-up for all three indices (**Figure 6**).

**Table 3:** Pairwise comparisons of time-dependent AUCs for ln CALLY, ln CLR, and ln CHR in predicting all-cause mortality at 1, 3, and 5 years.

During time	Comparison	AUC (ln CALLY/ln CLR)	AUC (comparison metric)	Z value	P
1-year					
	ln CALLY vs ln CLR	0.562	0.559	1.211	0.226
	ln CALLY vs ln CHR	0.562	0.540	1.600	0.110
	ln CLR vs ln CHR	0.559	0.540	1.394	0.163
3-year					
	ln CALLY vs ln CLR	0.603	0.598	2.358	0.018
	ln CALLY vs ln CHR	0.603	0.563	3.156	0.002
	ln CLR vs ln CHR	0.598	0.563	2.729	0.006
5-year					
	ln CALLY vs ln CLR	0.590	0.585	1.666	0.096
	ln CALLY vs ln CHR	0.590	0.581	0.683	0.495
	ln CLR vs ln CHR	0.585	0.581	0.303	0.761

AUC, area under the receiver operating characteristic curve; Z value, test statistic of the paired DeLong-type test based on the representation of the AUC estimator; P-value, statistical significance of the pairwise comparison.

### 3.7 Sensitivity analyses after multiple imputation

After multiple imputation, the full screened cohort of 798 patients was analyzed, including 633 survivors and 165 patients who died. The imputed cohort included 486 patients from Shenzhen People’s Hospital and 312 from Huidong People’s Hospital (**Supplementary Table 1**). Compared with survivors, patients who died were older (70.12 ± 12.32 vs. 58.55 ± 14.01 years, P < 0.0001). The distributions of hospital source, cause of ESKD, hypertension, diabetes, coronary heart disease, congestive heart failure, and history of stroke differed significantly between groups.

In the imputed cohort, patients who died had lower lymphocyte count and albumin and higher CRP levels. They also had lower CALLY and ln CALLY values and higher CLR, ln CLR, CHR, and ln CHR values than survivors. The distributions of ln CALLY, ln CLR, and ln CHR tertiles differed significantly by survival status. Deaths were concentrated in the lowest tertile of ln CALLY and in the highest tertiles of ln CLR and ln CHR.

Cox regression analyses in the imputed cohort were performed using the same model structure (**Supplementary Table 2**). Higher ln CALLY was associated with lower all-cause mortality risk (HR, 0.84; 95% CI, 0.77-0.91; P < 0.0001). Compared with Q1, Q2 and Q3 of ln CALLY were associated with lower mortality risk, with HRs of 0.58 (95% CI, 0.40-0.84; P = 0.004) and 0.56 (95% CI, 0.39-0.81; P = 0.002), respectively. The trend across tertiles was significant (P for trend = 0.001).

Higher ln CLR was associated with increased all-cause mortality risk (HR, 1.19; 95% CI, 1.09-1.29; P < 0.0001). The highest tertile of ln CLR was associated with higher mortality risk compared with the lowest tertile (HR, 1.68; 95% CI, 1.16-2.44; P = 0.01), whereas the middle tertile was not significant. The trend across tertiles was significant (P for trend = 0.005).

Higher ln CHR was also associated with increased all-cause mortality risk (HR, 1.16; 95% CI, 1.06-1.27; P = 0.001). Compared with Q1, Q3 of ln CHR was associated with higher mortality risk (HR, 1.54; 95% CI, 1.06-2.24; P = 0.02), whereas Q2 was not significant. The trend across tertiles remained significant (P for trend = 0.02).

### 3.8 Center-specific analyses

Center-specific Cox regression analyses were performed in Shenzhen People’s Hospital and Huidong People’s Hospital (**Supplementary Tables 3**, **4**). In Shenzhen People’s Hospital, the fully adjusted Model 3 showed that higher ln CALLY was associated with lower mortality risk (HR, 0.71; 95% CI, 0.60-0.85; P < 0.001), whereas higher ln CLR (HR, 1.42; 95% CI, 1.18-1.69; P < 0.001) and ln CHR (HR, 1.37; 95% CI, 1.12-1.67; P = 0.002) were associated with higher mortality risk. Compared with Q1, Q3 of ln CALLY was associated with lower mortality risk (HR, 0.28; 95% CI, 0.12-0.69; P = 0.01), while Q3 of ln CLR (HR, 4.09; 95% CI, 1.64-10.22; P = 0.003) and Q3 of ln CHR (HR, 3.34; 95% CI, 1.33-8.44; P = 0.01) were associated with higher mortality risk.

In Huidong People’s Hospital, the fully adjusted associations were not statistically significant. The HRs were 0.93 (95% CI, 0.80-1.07; P = 0.31) for ln CALLY, 1.08 (95% CI, 0.93-1.26; P = 0.30) for ln CLR, and 1.10 (95% CI, 0.94-1.29; P = 0.22) for ln CHR. Compared with Q1, Q3 HRs were 0.63 (95% CI, 0.33-1.20; P = 0.16) for ln CALLY, 1.30 (95% CI, 0.71-2.38; P = 0.40) for ln CLR, and 1.50 (95% CI, 0.81-2.81; P = 0.20) for ln CHR.

## 4 Discussion

In this retrospective two-center cohort study of patients undergoing MHD, three inflammation-related indices, namely CALLY, CLR, and CHR, were associated with all-cause mortality. Higher ln CALLY was associated with a lower risk of death, whereas higher ln CLR and ln CHR were associated with increased mortality risk. Kaplan-Meier analyses showed survival differences across tertiles of all three indices, and restricted cubic spline analyses showed significant overall associations without evidence of nonlinearity. Subgroup analyses showed no significant interactions across prespecified clinical strata. Sensitivity analyses after multiple imputation yielded similar results. Time-dependent ROC analyses showed modest discrimination for mortality prediction at 1, 3, and 5 years. These findings suggest that routinely available inflammation-related indices may provide information for mortality risk stratification in patients receiving MHD.

Our findings are broadly consistent with the expanding literature on inflammation-related indices in other disease settings. A lower CALLY index has been associated with poorer survival in hepatocellular carcinoma, distal cholangiocarcinoma, and colorectal cancer and, more recently, with higher cardiovascular event and mortality risks in chronic kidney disease (8–11). Elevated CLR has been reported to correlate with greater disease severity in acute pancreatitis, worse cardiac functional decline and survival in dilated cardiomyopathy, and poorer therapeutic outcomes in small cell lung cancer (6, 12, 13). Similarly, higher CHR has been linked to adverse outcomes after percutaneous coronary intervention, increased long-term mortality in the general population and individuals with cardiovascular-kidney-metabolic syndrome, and a higher risk of cardiometabolic multimorbidity (7, 14, 15). These observations support the concept that composite indices may capture shared biological processes involving inflammation, immune imbalance, metabolic dysfunction, and reduced physiological reserve.

The three indices examined in this study share an important biological foundation. Although CALLY, CLR, and CHR differ in mathematical structure, all are centered on CRP and therefore reflect inflammation-related risk. In patients undergoing MHD, chronic inflammatory activation is rarely isolated. It is closely linked to oxidative stress, endothelial injury, protein-energy wasting, immune dysfunction, infection susceptibility, and cardiovascular instability (2, 16, 17). These interconnected abnormalities are consistent with the malnutrition-inflammation-atherosclerosis framework and the malnutrition-inflammation complex syndrome described in dialysis populations (17). Therefore, the prognostic associations observed in our study may reflect not only the isolated effects of CRP, albumin, lymphocyte count, or HDL-C, but also the combined burden of inflammation, impaired nutritional reserve, immune dysregulation, and vascular injury.

At the molecular level, uremia and hemodialysis-related stress may activate several inflammatory pathways. Retention of uremic toxins, oxidative stress, recurrent exposure to dialysis membranes, vascular access-related inflammation, volume overload, and intermittent infection can activate innate immune responses and promote the release of IL-6, TNF-α, IL-1β, and other proinflammatory mediators (2, 16, 18). These cytokines can activate NF-κB signaling, increase the expression of endothelial adhesion molecules such as ICAM-1 and VCAM-1, and promote leukocyte adhesion, endothelial activation, and vascular inflammation (18, 19). Uremic toxins may also activate ROS, MAPK/NF-κB, aryl hydrocarbon receptor, and RAGE-related pathways, thereby aggravating endothelial dysfunction and vascular injury (18, 20). In parallel, inflammatory and oxidative pathways can facilitate vascular smooth muscle cell osteogenic transformation, vascular calcification, arterial stiffness, and atherosclerotic plaque instability. These processes may contribute to myocardial remodeling, impaired vascular compliance, hemodynamic vulnerability, and cardiovascular death. This mechanistic pathway provides a plausible explanation for why higher CRP-based indices were associated with adverse prognosis in our cohort.

CALLY may be particularly informative because it integrates inflammation, nutrition, and immune competence into a single index. A low CALLY value reflects elevated CRP, reduced albumin, and reduced lymphocyte count. These three abnormalities may interact biologically rather than act independently. Inflammation can suppress hepatic albumin synthesis through the acute-phase response, during which hepatic protein production shifts toward positive acute-phase proteins such as CRP and away from negative acute-phase proteins such as albumin (21). In patients receiving MHD, hypoalbuminemia therefore does not simply indicate inadequate nutritional intake. It may also reflect systemic inflammation, catabolic activation, fluid overload, albumin loss, and impaired physiological reserve. In addition, chronic inflammation, metabolic acidosis, insulin resistance, and uremic toxins can promote protein catabolism and skeletal muscle wasting through activation of the ubiquitin-proteasome system, caspase-3-dependent proteolysis, lysosomal pathways, myostatin signaling, and impaired PI3K/Akt/FoxO-mediated anabolic signaling (22, 23). These changes may accelerate protein-energy wasting, frailty, reduced functional reserve, hospitalization, and death. This may partly explain why lower ln CALLY was independently associated with higher mortality risk in our study.

The lymphocyte component of CALLY and CLR further links these indices to immune dysfunction. Patients with ESKD frequently show simultaneous immune activation and immune deficiency (24). Uremic toxins, oxidative stress, chronic inflammatory stimulation, and dialysis-related factors can impair both innate and adaptive immunity. Reported abnormalities include defective neutrophil and monocyte function, increased lymphocyte apoptosis, reduced naive T-cell output, expansion of differentiated or senescent T-cell populations, impaired antigen presentation, and weakened vaccine or antimicrobial responses. A low lymphocyte count may therefore indicate reduced immune reserve and impaired host defense. Clinically, this may increase susceptibility to bloodstream infection, pneumonia, sepsis, and poor recovery after acute inflammatory or cardiovascular events. CLR increases when CRP rises and lymphocyte count declines, thereby reflecting a state in which inflammatory activity exceeds immune capacity (24–26). Consequently, a low CALLY index and a high CLR may identify patients in whom inflammation and immune exhaustion coexist, resulting in greater vulnerability to infection-related and cardiovascular complications.

CHR may reflect another component of the same biological network: the interaction between inflammation and HDL-related vascular protection. HDL-C normally participates in reverse cholesterol transport and has antioxidant, anti-inflammatory, antiapoptotic, and endothelial-protective properties. However, in CKD and ESKD, HDL particles may become quantitatively reduced and qualitatively dysfunctional. Uremia and oxidative stress can alter HDL protein and lipid composition, enrich HDL with serum amyloid A, reduce apoA-I and paraoxonase-1 activity, impair cholesterol efflux, and weaken antioxidant and anti-inflammatory functions (27, 28). Dysfunctional HDL may fail to inhibit LDL oxidation, endothelial activation, and vascular inflammation and may even acquire proinflammatory properties. Experimental evidence further suggests that abnormal HDL in CKD can reduce endothelial nitric oxide availability through Toll-like receptor-2 activation, thereby impairing endothelial repair and promoting vascular dysfunction (29). Therefore, a high CHR may indicate a state in which inflammatory burden is high while HDL-mediated vascular protection is reduced. This mechanism may contribute to endothelial dysfunction, atherosclerosis, vascular calcification, cardiovascular instability, and ultimately higher mortality risk.

Taken together, these mechanisms suggest that CALLY, CLR, and CHR should be interpreted as integrated markers of inflammation-related vulnerability rather than simple laboratory ratios. A low CALLY index may capture the coexistence of systemic inflammation, protein-energy wasting, and impaired immune reserve. In contrast, high CLR and CHR may reflect inflammation-dominant states with reduced immune or lipid-related protective capacity. Although their mathematical directions differ, their biological interpretation is coherent: a more adverse inflammation-related profile is associated with a higher risk of death. These indices may therefore help identify patients with a greater burden of infection risk, cardiovascular injury, frailty, hospitalization, and reduced physiological resilience.

From a clinical perspective, these indices have practical advantages because they are derived from routine laboratory parameters and require no additional specialized testing. However, their role should be interpreted carefully. Patients undergoing MHD usually receive repeated laboratory testing within a single year, and CRP, albumin, lymphocyte count, and HDL-C may fluctuate over time because of infection, inflammation, nutritional intake, dialysis adequacy, volume status, vascular access events, and intercurrent illness (30). Therefore, a single baseline measurement should not be considered a definitive or permanent representation of long-term risk (30, 31). Rather, baseline CALLY, CLR, and CHR may serve as initial risk-stratification tools at cohort entry. They may help clinicians identify patients who require closer surveillance, nutritional assessment, screening for occult inflammation or infection, optimization of dialysis-related factors, and more intensive cardiovascular risk management. Their clinical and public health value may be greater when incorporated into longitudinal monitoring strategies. Future studies should evaluate repeated measurements, time-updated models, cumulative exposure, and trajectories of these indices to determine whether dynamic changes provide stronger prognostic information than baseline values alone.

Several limitations should be acknowledged. First, the present analysis was based primarily on baseline measurements. This approach does not capture intra-individual variability or longitudinal changes in CRP, albumin, lymphocyte count, HDL-C, and the derived indices during follow-up. Second, although multiple demographic, clinical, and hematological variables were adjusted for, residual confounding could not be completely excluded. Important factors such as dialysis adequacy, vascular access type, medication use, residual kidney function, inflammatory events during follow-up, and nutritional interventions were not incorporated into the current models. Third, patients with missing data required for index calculation were excluded from the complete-case analysis, which may have introduced selection bias, although sensitivity analyses after multiple imputation yielded consistent findings. Fourth, although hospital-specific sensitivity analyses showed broadly consistent directions of association across the two centers, these stratified analyses were constrained by the reduced sample size and the smaller number of events within each hospital. This limitation was particularly relevant when sequentially adjusted models were fitted separately in each center, as model stability may have been weakened. Therefore, the hospital-stratified findings should be interpreted as supportive sensitivity analyses rather than definitive center-specific evidence. Finally, this was a two-center study conducted in southern China, and the generalizability of our findings to other dialysis populations requires further validation in prospective multicenter cohorts.

## 5 Conclusion

In conclusion, CALLY, CLR, and CHR were significantly associated with all-cause mortality in patients undergoing MHD. Higher CALLY levels were associated with lower mortality risk, whereas higher CLR and CHR levels were associated with increased risk. As simple and readily available inflammation-related indices, they may offer practical value for prognostic assessment and risk stratification in this population. Further prospective multicenter studies are needed to validate these findings and clarify their clinical utility in long-term dialysis management.

## Data Availability

The raw data supporting the conclusions of this article will be made available by the authors, without undue reservation.
