# Supplementary material for: Associations of CALLY, CLR, and CHR with all-cause mortality in patients receiving maintenance hemodialysis: a two-center retrospective cohort study
Source: Front Endocrinol (Lausanne). 2026 Jul 6;17:1849614. doi: 10.3389/fendo.2026.1849614 (PMC13381296; doi:10.3389/fendo.2026.1849614)
Supplement: Supplementary file 1 [file Table1.docx]

Supplementary Table 1. Baseline characteristics of the imputed maintenance hemodialysis cohort stratified by survival status.

| Variable | Total (n=798) | Survivor (n=633) | Non-survivor (n=165) | p |
| --- | --- | --- | --- | --- |
| Hospital |  |  |  | 0.01 |
| Huidong | 312(39.10) | 233(36.81) | 79(47.88) |  |
| Shenzhen | 486(60.90) | 400(63.19) | 86(52.12) |  |
| Sex |  |  |  | 1.00 |
| Female | 299(37.47) | 237(37.44) | 62(37.58) |  |
| Male | 499(62.53) | 396(62.56) | 103(62.42) |  |
| Age | 60.94 ± 14.46 | 58.55 ± 14.01 | 70.12 ± 12.32 | <0.0001 |
| Dialysis age (months) | 43.22 ± 55.56 | 42.76 ± 54.89 | 45.01 ± 58.19 | 0.65 |
| During time (months) | 36.74 ± 23.44 | 40.41 ± 22.42 | 22.68 ± 22.00 | <0.0001 |
| Cause of ESKD |  |  |  | <0.0001 |
| Diabetic Nephropathy | 339(42.48) | 252(39.81) | 87(52.73) |  |
| Primary glomerulonephritis | 264(33.08) | 233(36.81) | 31(18.79) |  |
| Hypertensive nephropathy | 108(13.53) | 72(11.37) | 36(21.82) |  |
| Other | 87(10.90) | 76(12.01) | 11( 6.67) |  |
| Hypertension |  |  |  | <0.0001 |
| No | 230(28.82) | 205(32.39) | 25(15.15) |  |
| Yes | 568(71.18) | 428(67.61) | 140(84.85) |  |
| Diabetes |  |  |  | <0.001 |
| No | 539(67.54) | 446(70.46) | 93(56.36) |  |
| Yes | 259(32.46) | 187(29.54) | 72(43.64) |  |
| Coronary heart disease |  |  |  | <0.0001 |
| No | 602(75.44) | 500(78.99) | 102(61.82) |  |
| Yes | 196(24.56) | 133(21.01) | 63(38.18) |  |
| Congestive heart failure |  |  |  | <0.001 |
| No | 47( 5.89) | 27( 4.27) | 20(12.12) |  |
| Yes | 751(94.11) | 606(95.73) | 145(87.88) |  |
| Cerebral apoplexy history |  |  |  | <0.001 |
| No | 101(12.66) | 66(10.43) | 35(21.21) |  |
| Yes | 697(87.34) | 567(89.57) | 130(78.79) |  |
| WBC | 7.05 ± 2.82 | 7.08 ± 2.81 | 6.90 ± 2.84 | 0.44 |
| RBC | 3.40 ± 0.83 | 3.41 ± 0.84 | 3.37 ± 0.75 | 0.56 |
| HB | 96.75 ± 21.87 | 96.97 ± 22.48 | 95.92 ± 19.40 | 0.55 |
| PLT | 203.09 ± 72.78 | 203.81 ± 73.25 | 200.32 ± 71.09 | 0.58 |
| LYM | 1.23 ± 0.54 | 1.27 ± 0.55 | 1.08 ± 0.48 | <0.0001 |
| ALB | 37.35 ± 5.48 | 37.67 ± 5.61 | 36.11 ± 4.78 | <0.001 |
| CRP | 2.76 (1.01, 6.26) | 2.45 (1.01, 6.26) | 3.80 (1.30, 12.88) | 0.001 |
| HDL-C | 1.03 ± 0.35 | 1.04 ± 0.34 | 1.01 ± 0.38 | 0.28 |
| Composite inflammation-related indices |  |  |  |  |
| CALLY | 1.49 (0.57, 4.23) | 1.70 (0.65, 4.81) | 0.90 (0.28, 3.20) | <0.001 |
| ln CALLY | 0.40 (-0.56, 1.44) | 0.53 (-0.43, 1.57) | -0.10 (-1.29, 1.16) | <0.001 |
| ln CALLY Group |  |  |  | <0.001 |
| Q1 | 266(33.33) | 189(29.86) | 77(46.67) |  |
| Q2 | 266(33.33) | 222(35.07) | 44(26.67) |  |
| Q3 | 266(33.33) | 222(35.07) | 44(26.67) |  |
| CHR | 2.71 (0.92, 7.90) | 2.50 (0.85, 6.45) | 3.97 (1.20, 14.67) | <0.001 |
| ln CHR | 1.00 (-0.08, 2.07) | 0.92 (-0.16, 1.86) | 1.38 (0.18, 2.69) | <0.001 |
| ln CHR Group |  |  |  | <0.01 |
| Q1 | 266(33.33) | 221(34.91) | 45(27.27) |  |
| Q2 | 266(33.33) | 219(34.60) | 47(28.48) |  |
| Q3 | 266(33.33) | 193(30.49) | 73(44.24) |  |
| CLR | 2.42 (0.85, 6.44) | 2.18 (0.73, 5.59) | 4.06 (1.20, 13.44) | <0.001 |
| ln CLR | 0.89 (-0.16, 1.86) | 0.78 (-0.31, 1.72) | 1.40 (0.18, 2.60) | <0.001 |
| ln CLR Group |  |  |  | <0.01 |
| Q1 | 266(33.33) | 222(35.07) | 44(26.67) |  |
| Q2 | 266(33.33) | 220(34.76) | 46(27.88) |  |
| Q3 | 266(33.33) | 191(30.17) | 75(45.45) |  |
| Abbreviations: ESKD, End-Stage Renal Disease; WBC, white blood cell; RBC, Red Blood Cell; HB, Hemoglobin; PLT, Platelets; LYM, Lymphocyte; ALB, Albumin; CRP, C-Reactive Protein; HDL-C, High-Density Lipoprotein Cholesterol; CALLY, C-reactive protein to Albumin Lymphocyte ratio; CLR, CRP to Lymphocyte ratio; CHR, CRP to HDL-C ratio. | | | | |

Supplementary Table 2. Cox regression analyses of associations between log-transformed inflammation-related indices and all-cause mortality after multiple imputation.

| **Character** | **Crude model** | | **Model 1** | | **Model 2** | | **Model 3** | |
| --- | --- | --- | --- | --- | --- | --- | --- | --- |
|  | **95%CI** | **P** | **95%CI** | **P** | **95%CI** | **P** | **95%CI** | **P** |
| **ln CALLY** | 0.84(0.77,0.91) | <0.0001 | 0.88(0.81,0.96) | 0.003 | 0.88(0.81,0.96) | 0.005 | 0.84(0.77,0.91) | <0.0001 |
| **ln CALLY group** |  |  |  |  |  |  |  |  |
| Q1 | ref |  | ref |  | ref |  | ref |  |
| Q2 | 0.58(0.40,0.84) | 0.004 | 0.57(0.39,0.83) | 0.004 | 0.58(0.39,0.84) | 0.004 | 0.58(0.40,0.84) | 0.004 |
| Q3 | 0.56(0.39,0.81) | 0.002 | 0.62(0.43,0.91) | 0.01 | 0.65(0.44,0.94) | 0.02 | 0.56(0.39,0.81) | 0.002 |
| p for trend |  | 0.001 |  | 0.01 |  | 0.01 |  | 0.001 |
| **ln CLR** | 1.19(1.09,1.29) | <0.0001 | 1.14(1.04,1.24) | 0.003 | 1.14(1.04,1.24) | 0.004 | 1.19(1.09,1.29) | <0.0001 |
| **ln CLR group** |  |  |  |  |  |  |  |  |
| Q1 | ref |  | ref |  | ref |  | ref |  |
| Q2 | 1.06(0.70,1.60) | 0.78 | 0.99(0.65,1.51) | 0.98 | 0.96(0.63,1.46) | 0.86 | 1.06(0.70,1.60) | 0.78 |
| Q3 | 1.68(1.16,2.44) | 0.01 | 1.52(1.04,2.22) | 0.03 | 1.47(1.01,2.14) | 0.04 | 1.68(1.16,2.44) | 0.01 |
| p for trend |  | 0.005 |  | 0.02 |  | 0.03 |  | 0.005 |
| **ln CHR** | 1.16(1.06,1.27) | 0.001 | 1.12(1.02,1.22) | 0.01 | 1.12(1.02,1.23) | 0.01 | 1.16(1.06,1.27) | 0.001 |
| **ln CHR group** |  |  |  |  |  |  |  |  |
| Q1 | ref |  | ref |  | ref |  | ref |  |
| Q2 | 1.08(0.72,1.63) | 0.70 | 1.11(0.73,1.67) | 0.63 | 1.07(0.71,1.61) | 0.76 | 1.08(0.72,1.63) | 0.70 |
| Q3 | 1.54(1.06,2.24) | 0.02 | 1.53(1.05,2.23) | 0.03 | 1.51(1.03,2.20) | 0.03 | 1.54(1.06,2.24) | 0.02 |
| p for trend |  | 0.02 |  | 0.02 |  | 0.03 |  | 0.02 |
| Crude model: Composite Inflammation-Related Indices. | | | | | | | | |
| Model 1: Crude model further adjusted age, Sex, Dialysis age, Cause of ESKD. | | | | | | | | |
| Model 2: Crude model further adjusted age, Sex, Dialysis age, Cause of ESKD, Hypertension, Diabetes, Coronary heart disease, Congestive heart failure, Cerebral apoplexy history | | | | | | | | |
| Model 3: Crude model further adjusted age, Sex, Dialysis age, Cause of ESKD, Hypertension, Diabetes, Coronary heart disease, Congestive heart failure, Cerebral apoplexy history, WBC, RBC, HB, PLT | | | | | | | | |

Supplementary Table 3. Hospital-specific sensitivity analysis of associations between log-transformed inflammation-related indices and all-cause mortality in patients from Shenzhen People’s Hospital.

| **Character** | **Crude model** | | **Model 1** | | **Model 2** | | **Model 3** | |
| --- | --- | --- | --- | --- | --- | --- | --- | --- |
|  | **95%CI** | **P** | **95%CI** | **P** | **95%CI** | **P** | **95%CI** | **P** |
| **ln CALLY** | 0.76(0.66,0.87) | <0.001 | 0.81(0.70,0.94) | 0.004 | 0.78(0.67, 0.91) | 0.001 | 0.71(0.60, 0.85) | <0.001 |
| **ln CALLY group** |  |  |  |  |  |  |  |  |
| Q1 | ref |  | ref |  | ref |  | ref |  |
| Q2 | 0.68(0.35,1.29) | 0.24 | 0.69(0.35,1.35) | 0.28 | 0.61(0.30, 1.23) | 0.17 | 0.52(0.24, 1.12) | 0.10 |
| Q3 | 0.35(0.16,0.75) | 0.01 | 0.41(0.18,0.91) | 0.03 | 0.34(0.15, 0.78) | 0.01 | 0.28(0.12, 0.69) | 0.01 |
| p for trend |  | 0.01 |  | 0.03 |  | 0.01 |  | 0.005 |
| **ln CLR** | 1.33(1.15,1.54) | <0.001 | 1.24(1.07,1.44) | 0.005 | 1.28(1.10, 1.50) | 0.001 | 1.42(1.18, 1.69) | <0.001 |
| **ln CLR group** |  |  |  |  |  |  |  |  |
| Q1 | ref |  | ref |  | ref |  | ref |  |
| Q2 | 2.3(0.98,5.38) | 0.06 | 2.07(0.86,4.97) | 0.10 | 2.11(0.87, 5.12) | 0.10 | 2.18(0.89, 5.31) | 0.09 |
| Q3 | 3.27(1.47,7.29) | 0.004 | 2.82(1.22,6.53) | 0.02 | 3.37(1.41, 8.07) | 0.01 | 4.09(1.64,10.22) | 0.003 |
| p for trend |  | 0.003 |  | 0.02 |  | 0.01 |  | 0.002 |
| **ln CHR** | 1.32(1.13,1.55) | <0.001 | 1.21(1.03,1.42) | 0.02 | 1.23(1.05, 1.45) | 0.01 | 1.37(1.12, 1.67) | 0.002 |
| **ln CHR group** |  |  |  |  |  |  |  |  |
| Q1 | ref |  | ref |  | ref |  | ref |  |
| Q2 | 1.82(0.77,4.29) | 0.17 | 1.51(0.63,3.61) | 0.35 | 1.68(0.69, 4.08) | 0.25 | 1.69(0.69, 4.15) | 0.25 |
| Q3 | 3.21(1.45,7.14) | 0.004 | 2.32(1.01,5.35) | 0.05 | 2.75(1.15, 6.60) | 0.02 | 3.34(1.33, 8.44) | 0.01 |
| p for trend |  | 0.003 |  | 0.04 |  | 0.02 |  | 0.01 |
| Crude model: Composite Inflammation-Related Indices. | | | | | | | | |
| Model 1: Crude model further adjusted age, Sex, Dialysis age, Cause of ESKD. | | | | | | | | |
| Model 2: Crude model further adjusted age, Sex, Dialysis age, Cause of ESKD, Hypertension, Diabetes, Coronary heart disease, Congestive heart failure, Cerebral apoplexy history | | | | | | | | |
| Model 3: Crude model further adjusted age, Sex, Dialysis age, Cause of ESKD, Hypertension, Diabetes, Coronary heart disease, Congestive heart failure, Cerebral apoplexy history, WBC, RBC, HB, PLT | | | | | | | | |

Supplementary Table 4. Hospital-specific sensitivity analysis of associations between log-transformed inflammation-related indices and all-cause mortality in patients from Huidong People’s Hospital.

| **Character** | **Crude model** | | **Model 1** | | **Model 2** | | **Model 3** | |
| --- | --- | --- | --- | --- | --- | --- | --- | --- |
|  | **95%CI** | **P** | **95%CI** | **P** | **95%CI** | **P** | **95%CI** | **P** |
| **ln CALLY** | 0.83(0.73,0.94) | 0.004 | 0.9(0.79,1.03) | 0.14 | 0.91(0.79,1.04) | 0.16 | 0.93(0.80,1.07) | 0.31 |
| **ln CALLY group** |  |  |  |  |  |  |  |  |
| Q1 | ref |  | ref |  | ref |  | ref |  |
| Q2 | 0.67(0.40,1.13) | 0.13 | 0.83(0.49,1.41) | 0.50 | 0.83(0.48,1.42) | 0.49 | 0.88(0.50,1.55) | 0.66 |
| Q3 | 0.42(0.24,0.76) | 0.004 | 0.58(0.32,1.07) | 0.08 | 0.59(0.32,1.09) | 0.09 | 0.63(0.33,1.20) | 0.16 |
| p for trend |  | 0.003 |  | 0.08 |  | 0.09 |  | 0.17 |
| **ln CLR** | 1.2(1.06,1.37) | 0.005 | 1.11(0.97,1.27) | 0.14 | 1.11(0.96,1.27) | 0.16 | 1.08(0.93,1.26) | 0.30 |
| **ln CLR group** |  |  |  |  |  |  |  |  |
| Q1 | ref |  | ref |  | ref |  | ref |  |
| Q2 | 1.15(0.63,2.11) | 0.65 | 0.98(0.52,1.83) | 0.94 | 0.98(0.52,1.85) | 0.96 | 0.97(0.52,1.84) | 0.94 |
| Q3 | 1.97(1.14,3.42) | 0.02 | 1.38(0.78,2.44) | 0.26 | 1.38(0.77,2.47) | 0.27 | 1.3(0.71,2.38) | 0.40 |
| p for trend |  | 0.01 |  | 0.22 |  | 0.24 |  | 0.37 |
| **ln CHR** | 1.17(1.03,1.34) | 0.02 | 1.11(0.97,1.28) | 0.14 | 1.12(0.97,1.29) | 0.13 | 1.1(0.94,1.29) | 0.22 |
| **ln CHR group** |  |  |  |  |  |  |  |  |
| Q1 | ref |  | ref |  | ref |  | ref |  |
| Q2 | 1.38(0.75,2.51) | 0.30 | 1.25(0.68,2.31) | 0.48 | 1.2(0.64,2.24) | 0.58 | 1.2(0.63,2.27) | 0.57 |
| Q3 | 2.04(1.16,3.57) | 0.01 | 1.57(0.89,2.80) | 0.12 | 1.57(0.87,2.81) | 0.13 | 1.5(0.81,2.81) | 0.20 |
| p for trend |  | 0.01 |  | 0.12 |  | 0.12 |  | 0.19 |
| Crude model: Composite Inflammation-Related Indices. | | | | | | | | |
| Model 1: Crude model further adjusted age, Sex, Dialysis age, Cause of ESKD. | | | | | | | | |
| Model 2: Crude model further adjusted age, Sex, Dialysis age, Cause of ESKD, Hypertension, Diabetes, Coronary heart disease, Congestive heart failure, Cerebral apoplexy history | | | | | | | | |
| Model 3: Crude model further adjusted age, Sex, Dialysis age, Cause of ESKD, Hypertension, Diabetes, Coronary heart disease, Congestive heart failure, Cerebral apoplexy history, WBC, RBC, HB, PLT | | | | | | | | |
